# Making topological protein links using enzymatic reactions

**DOI:** 10.1093/nsr/nwae071

**Published:** 2024-04-03

**Authors:** Roger Castells-Graells, Todd O Yeates

**Affiliations:** Department of Chemistry and Biochemistry, University of California, USA; UCLA-DOE Institute for Genomics and Proteomics, USA; Department of Chemistry and Biochemistry, University of California, USA; UCLA-DOE Institute for Genomics and Proteomics, USA

Proteins are exceedingly complex molecules though, at their heart, they are essentially linear polymers. Pull at their ends and one obtains an extended backbone. While that is true in most cases, some natural proteins have evolved elaborations that complicate the picture. These modifications typically stabilize a natively folded conformation and prevent its unfolding. Numerous structural and chemical mechanisms for embellishing the protein backbone have been elucidated, ranging from simple disulfide bonds to much more complex topological features. The diversity of naturally evolved variations speaks to their functional importance in stabilizing proteins against chemical, thermal or physical unfolding forces.

Certain types of protein backbone variation, such as disulfide bonds, are readily understood in terms of local structure. Other potentially stabilizing features can only be understood in terms of the overall global path of the protein backbone and its topological entanglements. For example, there are rare cases in which the path of a protein backbone forms a knot in 3D space. In other rare examples, two protein chains are cyclized by disulfide bonds in a way that links them and makes them inseparable [1,2].

Whether in the context of a protein backbone or purely mathematical curves in space, the global nature of knotting and linking makes it non-trivial to identify such topologically complex structures [3]. Indeed, the first deeply knotted protein backbone was discovered [4] only after the initial structural report. The same considerations—global vs. local dependence—make the engineering of knotted and/or interlinked proteins uniquely challenging. A first demonstration of designed protein knotting came from fusing the monomers of a naturally homodimeric protein; the two subunits were naturally twisted about each other in a way that made the fused form fortuitously knotted [5]. On the related problem of forming a link or catenane between two protein chains, this was first achieved in a similar fashion by connecting the ends of the entwined chains of a homodimeric p53 protein domain [6].

**Figure 1.**
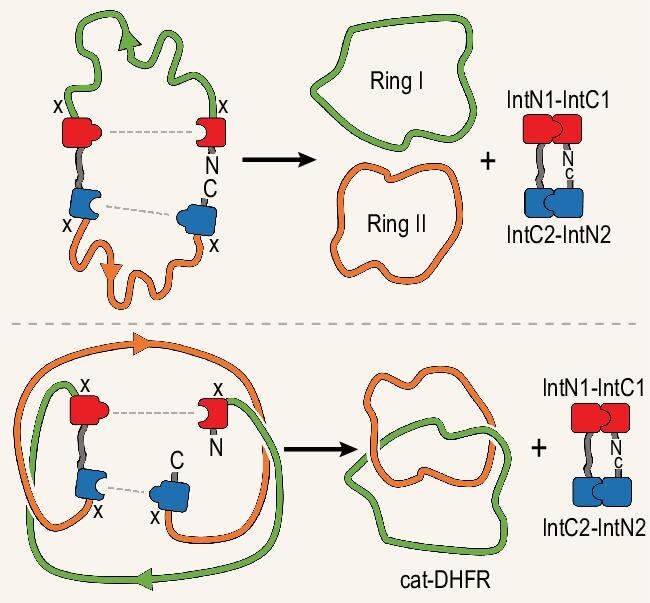
A schematic diagram showing the creation of two closed rings (right) from two intein-based splicing reactions on a linear protein backbone (left). The paths of the protein backbones are illustrated as smoothed curves without folded protein characteristics in order to clarify the underlying topological relationships. The top panel illustrates the expected outcome—unlinked rings—from an arbitrary choice of protein as the framework. The bottom panel illustrates the case of linked rings obtained for special choices of the underlying protein framework, which was the enzyme dihydrofolate reductase described by Fang *et al*. [8]. The dashed lines indicate new connections formed by the intein ligations, with cleavages at positions marked by ‘X’.

The creation of topologically complex protein backbones—links or knots—of diverse form is possible if additional backbone connections can be made and broken, all while the backbone is held in a specific 3D configuration. Building upon considerable prior research [7], in a recent report, Fang *et al*. [8] demonstrate this kind of backbone ‘rewiring’ through clever application of chemical biology tools, in combination with approximate 3D modeling. The researchers identified the enzyme dihydrofolate reductase (DHFR) as one in which the native 3D structure could support a rewiring process leading to two closed interlinked chains, known as a catenane. The process involves two enzymatic reactions catalysed by orthogonal inteins—self-reacting splicing motifs—engineered into the starting gene construct. Each intein reaction closes a ring between one of the original chain termini and a position on the chain interior (Fig. 1). Together, the two ring closures release the intein motifs, leaving the DHFR catenane as the product, which retains enzymatic activity in its rewired form. The autocatalytic feature of the construct makes it an effectively

one-pot synthetic system in the cell. The authors go on to demonstrate superior thermal stability of the catenane version of the novel DHFR compared with its natural linear form. This dual-intein catenane process adds a new twist to the field of engineering topologically complex proteins.

This latest protein engineering achievement is a timely one, dovetailing with powerful new advances in machine-learning and artificial intelligence tools for protein engineering [9]. Previous successes in designing topologically complex proteins, the present work included, have emerged from human insight into how knotted or linked curves might be generated. Even more complex structures and topological variations are likely to flow in the future from creative applications of rapidly improving computer algorithms [10]. Topological protein forms of the type presented here should be enticing targets for these new computational tools.

***Conflict of interest statement*.** None declared.
